# Novel one-step deployment of Y-shaped metal stent: no more stent-in-stent or side-by-side

**DOI:** 10.1055/a-2783-2573

**Published:** 2026-02-13

**Authors:** Kazumasa Nagai, Takao Itoi, Ryosuke Tonozuka, Shuntaro Mukai, Akio Katanuma, Shomei Ryozawa, Yoshinobu Okabe

**Affiliations:** 1 13112 Department of Gastroenterology and Hepatology, Tokyo Medical University, Tokyo, Japan; 2 92187 Department of Gastroenterology and Hepatology, Sapporo Medical University School of Medicine, Sapporo, Japan; 3 183786 Department of Gastroenterology, Saitama Medical University International Medical Center, Saitama, Japan; 4 26333 Division of Gastroenterology, Department of Internal Medicine, Kurume University School of Medicine, Kurume, Japan

**Fig. 1:**
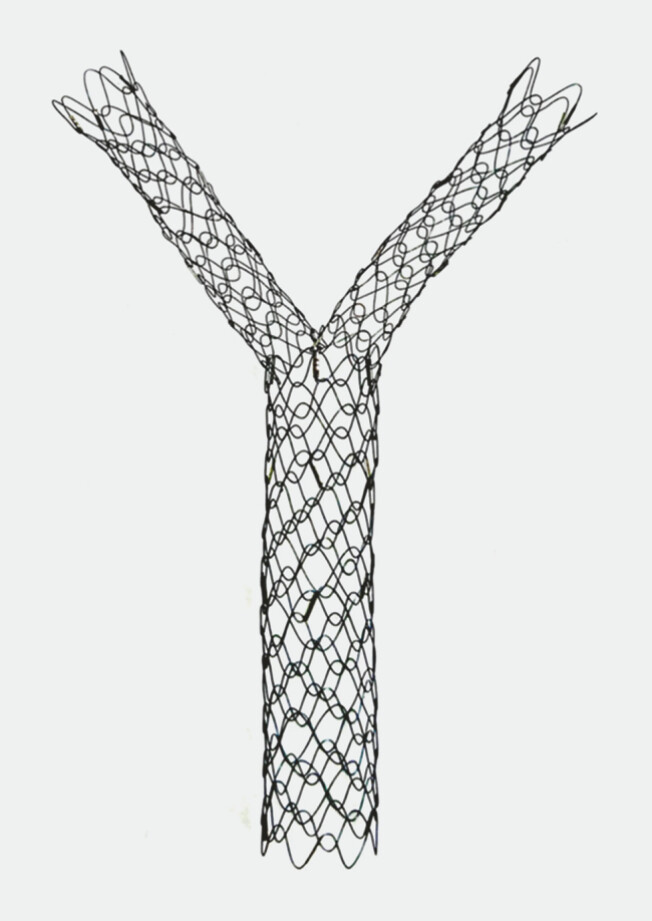
A newly developed Y-shaped metal stent (YAMS).

**Fig. 2:**
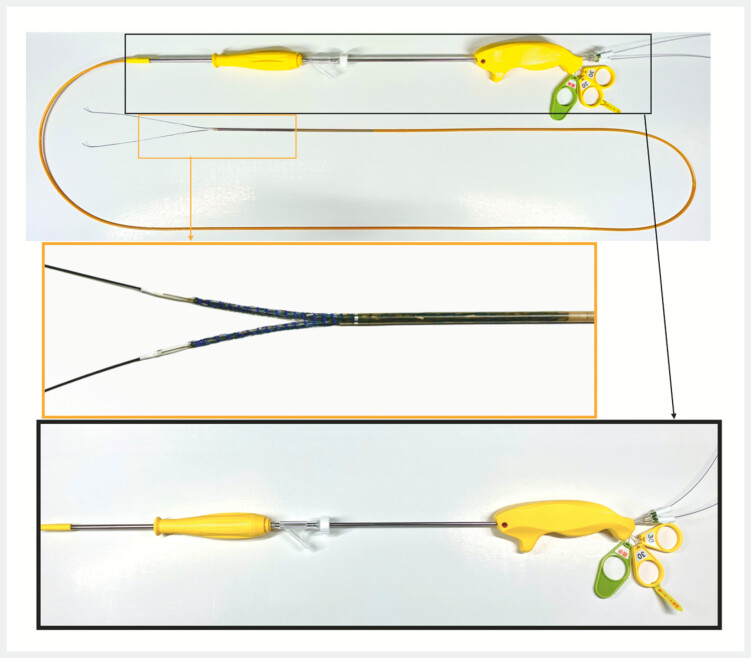
Y-shaped Metal Stent (YAMS) has a bifurcated distal tip and is equipped with three control levers at the proximal end of the delivery system to facilitate Y-shaped deployment.

Biliary drainage is an essential procedure for malignant hilar biliary obstruction from hilar cholangiocarcinoma. Multiple plastic stent placement or self-expandable metal stent deployment using stent-in-stent or side-by-side techniques are commonly employed; however, these procedures can be technically challenging, and adequate drainage is not always achieved 1 2 3 . We report the world’s first clinical application of a newly developed system that enables one-step placement of a Y-shaped metal stent (YAMS) ( Fig. 1 ). The YAMS has a bifurcated distal tip and is deployed into a Y-shape by sequentially pulling three control levers at the proximal end of the delivery system ( Fig. 2 , Video 1 ).

Tabletop demonstration and clinical deployment of the Y-shaped Metal Stent (YAMS).Video 1

**Fig. 3:**
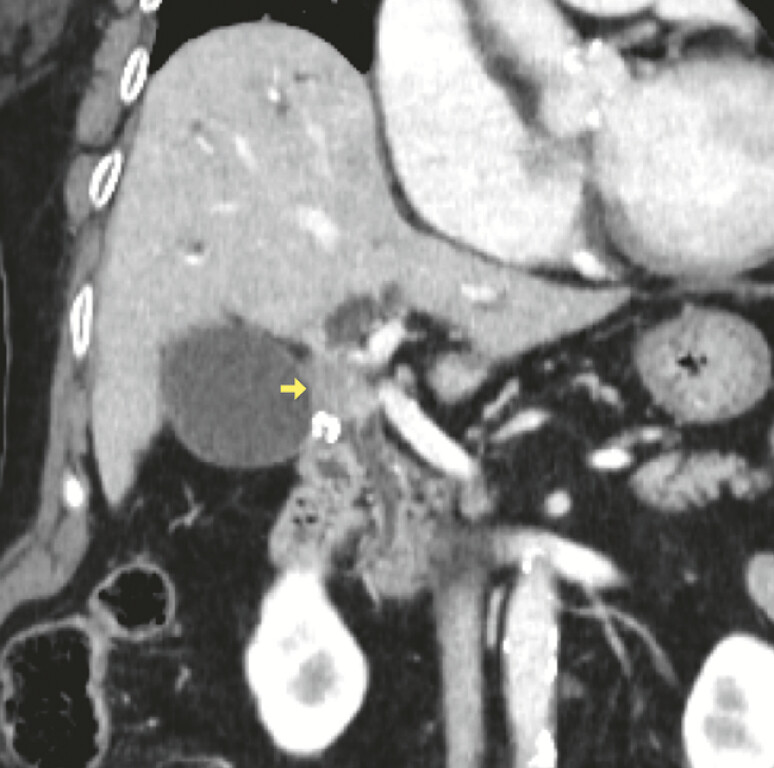
CT revealed a mass in the hilar bile duct region (yellow arrow).

**Fig. 4:**
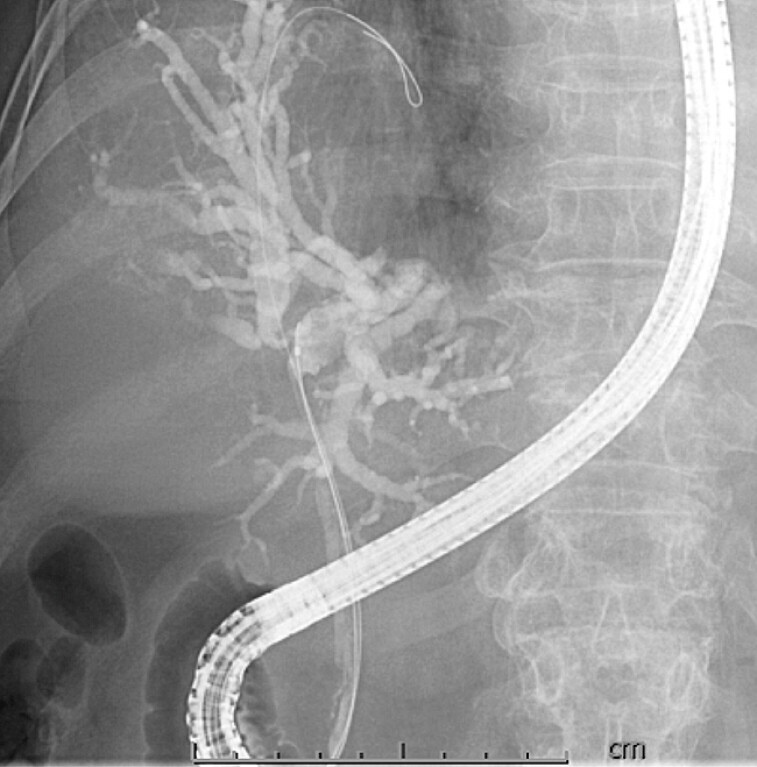
On cholangiography, a hilar biliary stricture was identified and classified as Bismuth type II.

**Fig. 5:**
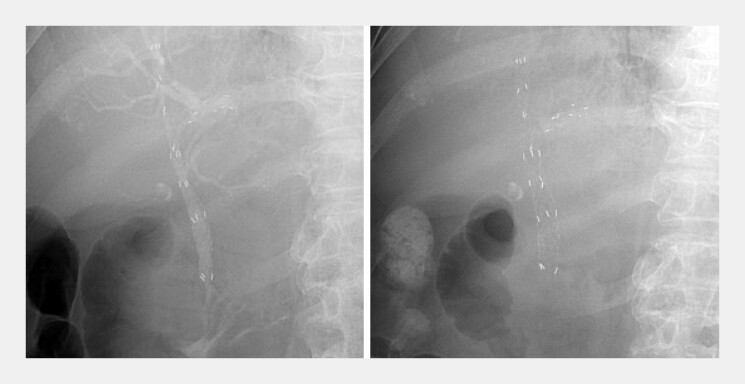
An uncovered Y-shaped Metal Stent (YAMS) was successfully deployed across the hilar stricture, extending into both hepatic ducts ( **a** ). Follow-up X-ray obtained two days later confirmed full expansion of the YAMS ( **b** ).

An 85-year-old woman was diagnosed with hilar cholangiocarcinoma ( Fig. 3 ) and managed with best supportive care. She presented with a Bismuth type II malignant biliary obstruction ( Fig. 4 ). After biliary decompression with endoscopic nasobiliary drainage, one-step YAMS placement was attempted. Cholangiography confirmed the extent of the stricture, and 0.025-inch guidewires were placed in the right and left hepatic ducts. The stent delivery sheath (9.4 Fr, 1900 mm in length, compatible with a 0.025-inch guidewire) was advanced to the target site. Once fluoroscopy confirmed the bifurcated stent tip directed toward the right and left hepatic ducts, stent deployment was initiated. An uncovered YAMS was successfully placed across the stricture, extending into both hepatic ducts ( Fig. 5 **a** , Video 1 ). The YAMS selected measured 10 mm × 40 mm at the common bile duct and 6 mm × 30 mm at each hepatic duct. Follow-up X-ray performed two days later confirmed full expansion of the YAMS ( Fig. 5 **b** ). The patient achieved effective drainage and was discharged the following day with resolution of jaundice. This novel stent design enables simultaneous bilateral drainage in one step while preserving biliary anatomy without overdilation. We consider this a groundbreaking device and report its first clinical use.

Endoscopy_UCTN_Code_TTT_1AR_2AZ
